# Supplementary figures and images for: Multi-Omic Analysis of Esophageal Adenocarcinoma Uncovers Candidate Therapeutic Targets and Cancer-Selective Posttranscriptional Regulation
Source: Mol Cell Proteomics. 2024 Apr 9;23(6):100764. doi: 10.1016/j.mcpro.2024.100764 (PMC11245951; doi:10.1016/j.mcpro.2024.100764)

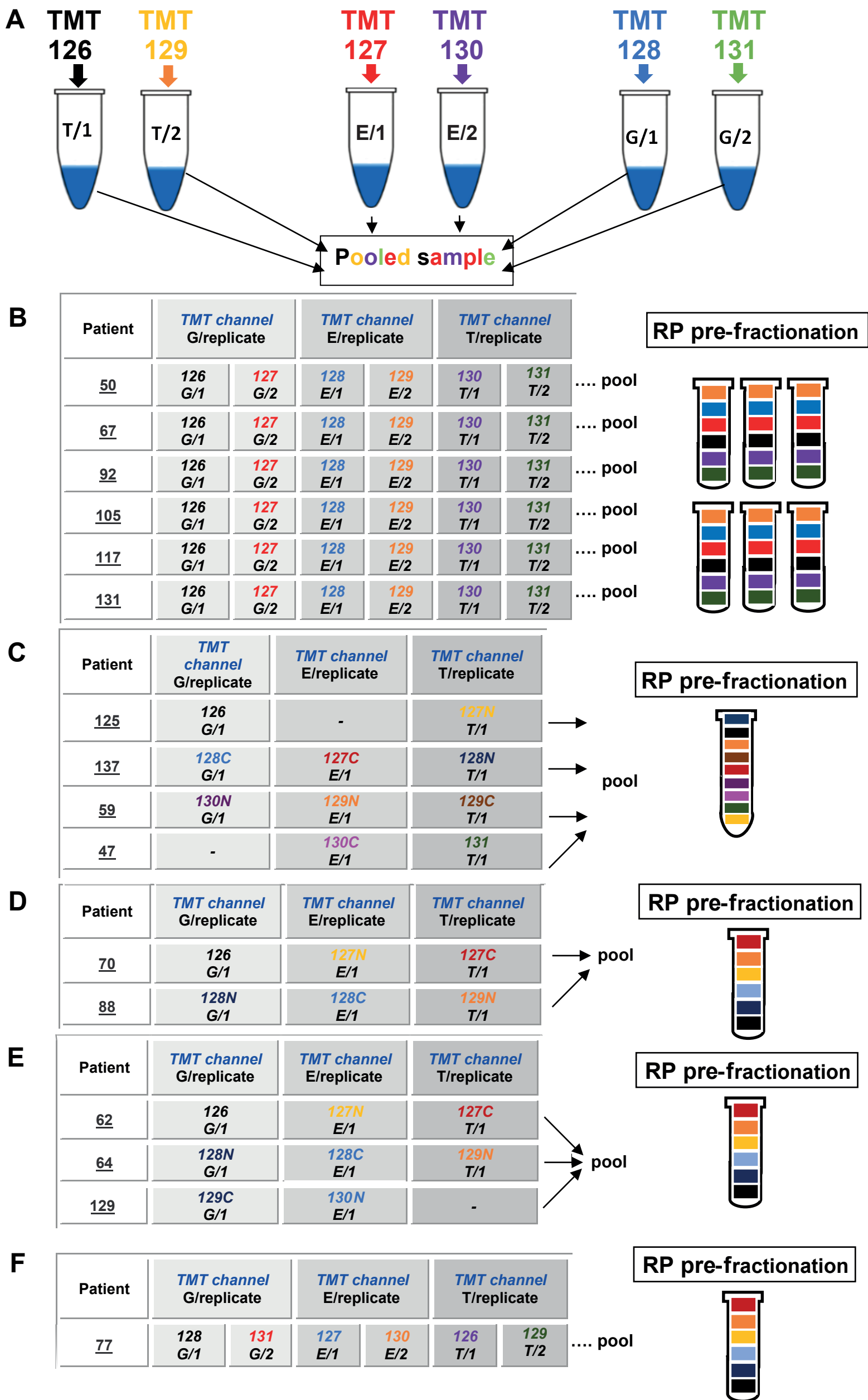

Supplement: Supplemental Figure S1 [file mmc1.pdf]

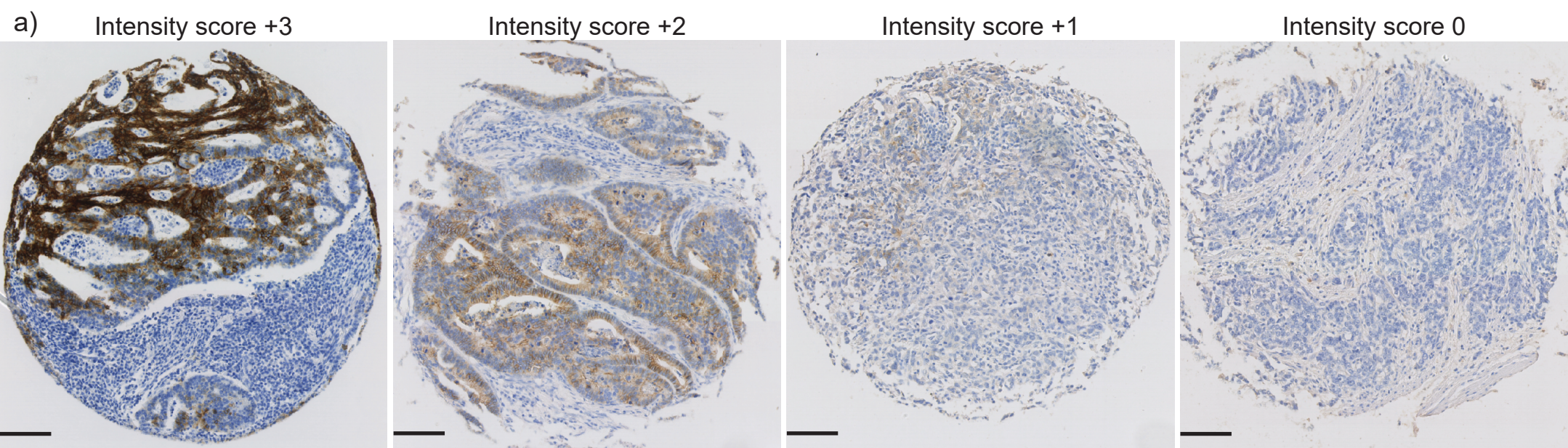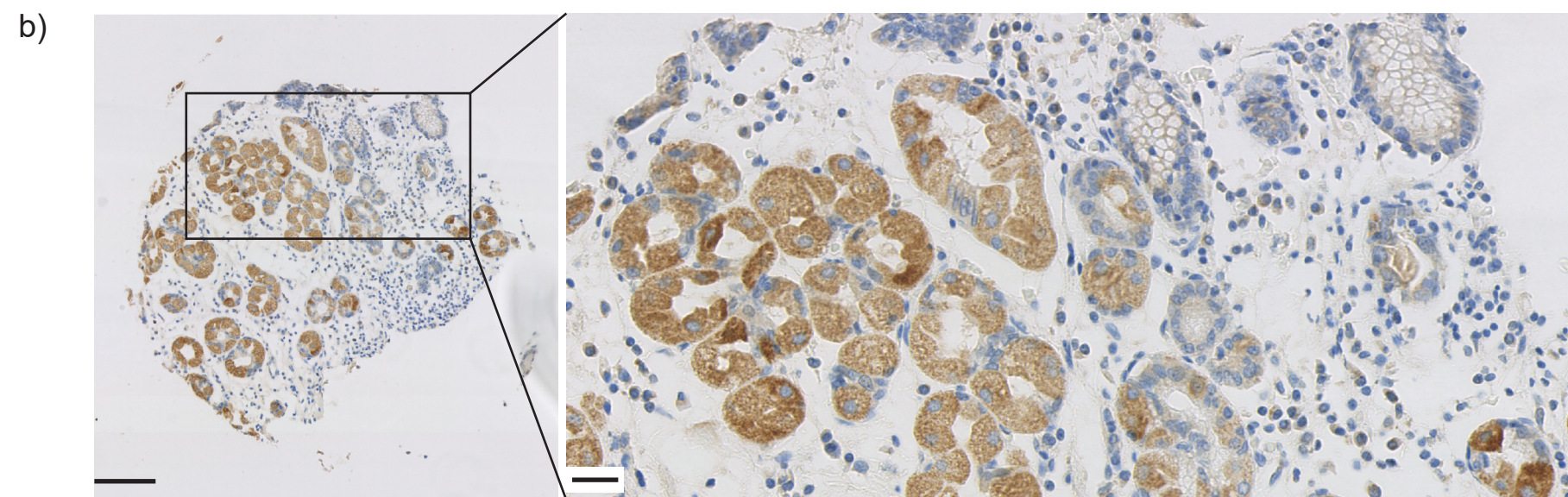

Supplement: Supplemental Figure S2 [file mmc2.pdf]

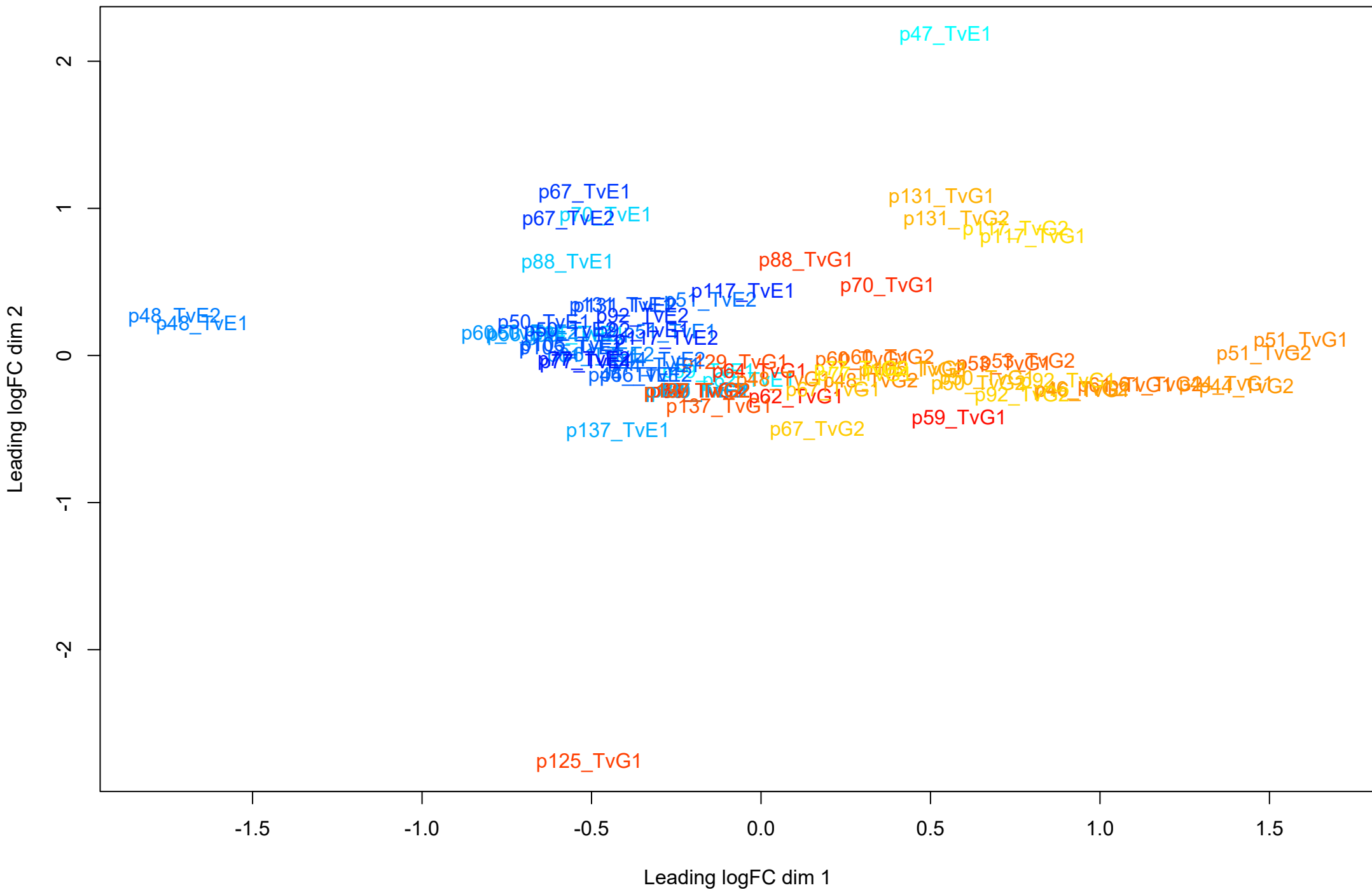

Supplement: Supplemental Figure S3 [file mmc3.pdf]

a) Gastric

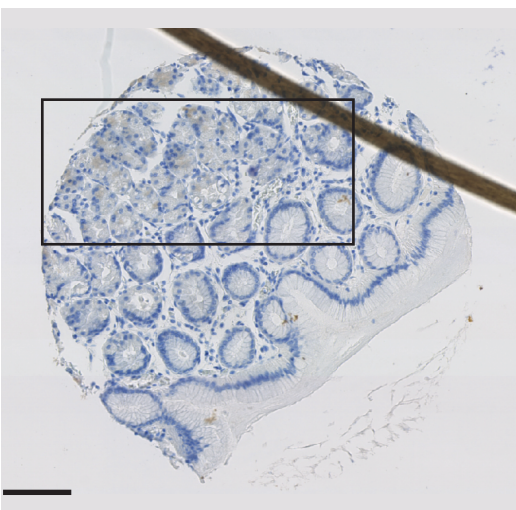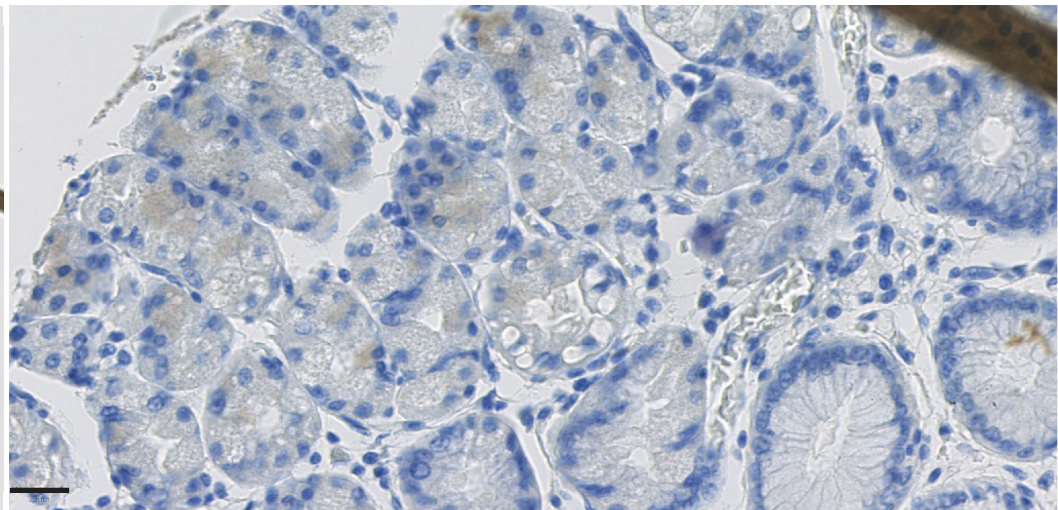

b) Squamous

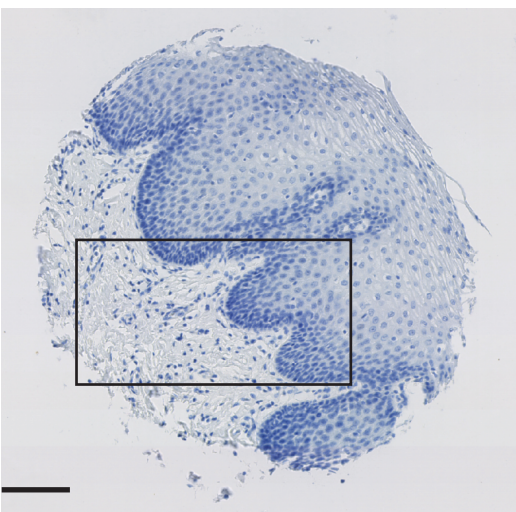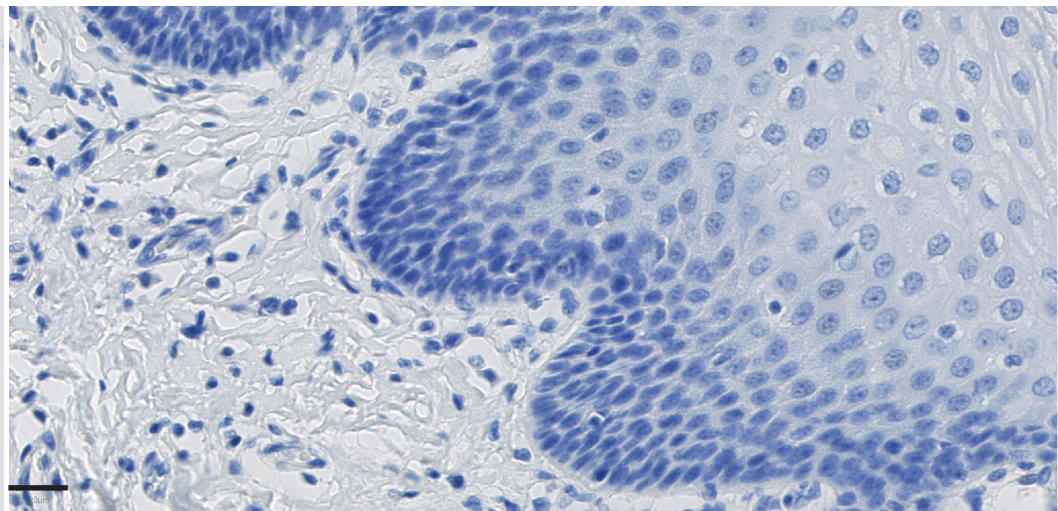

c) Uninvolved node

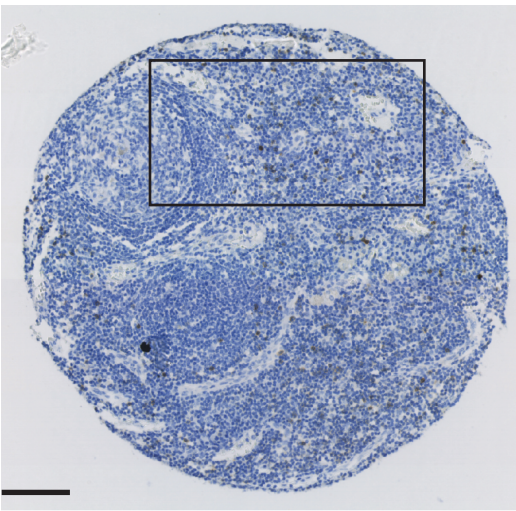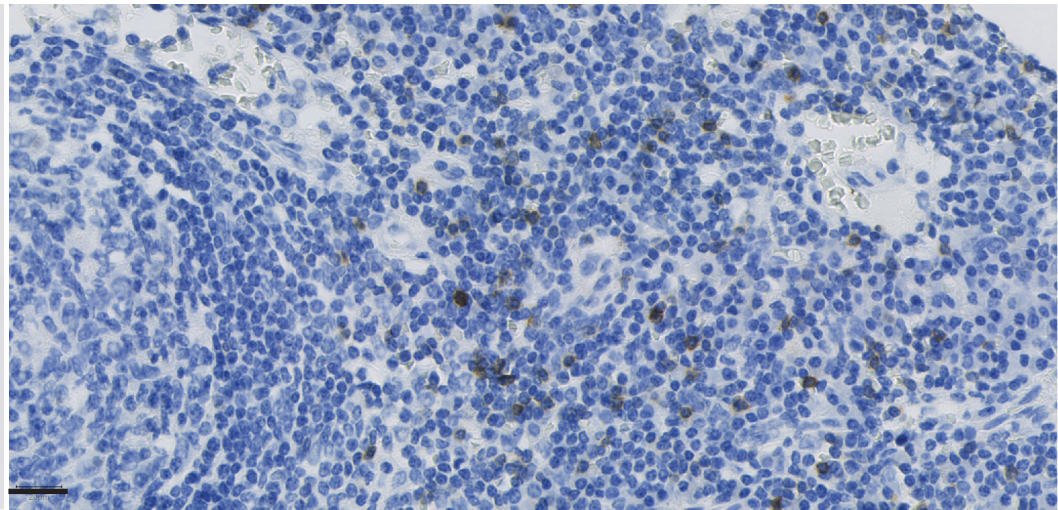

Supplement: Supplemental Figure S5 [file mmc4.pdf]

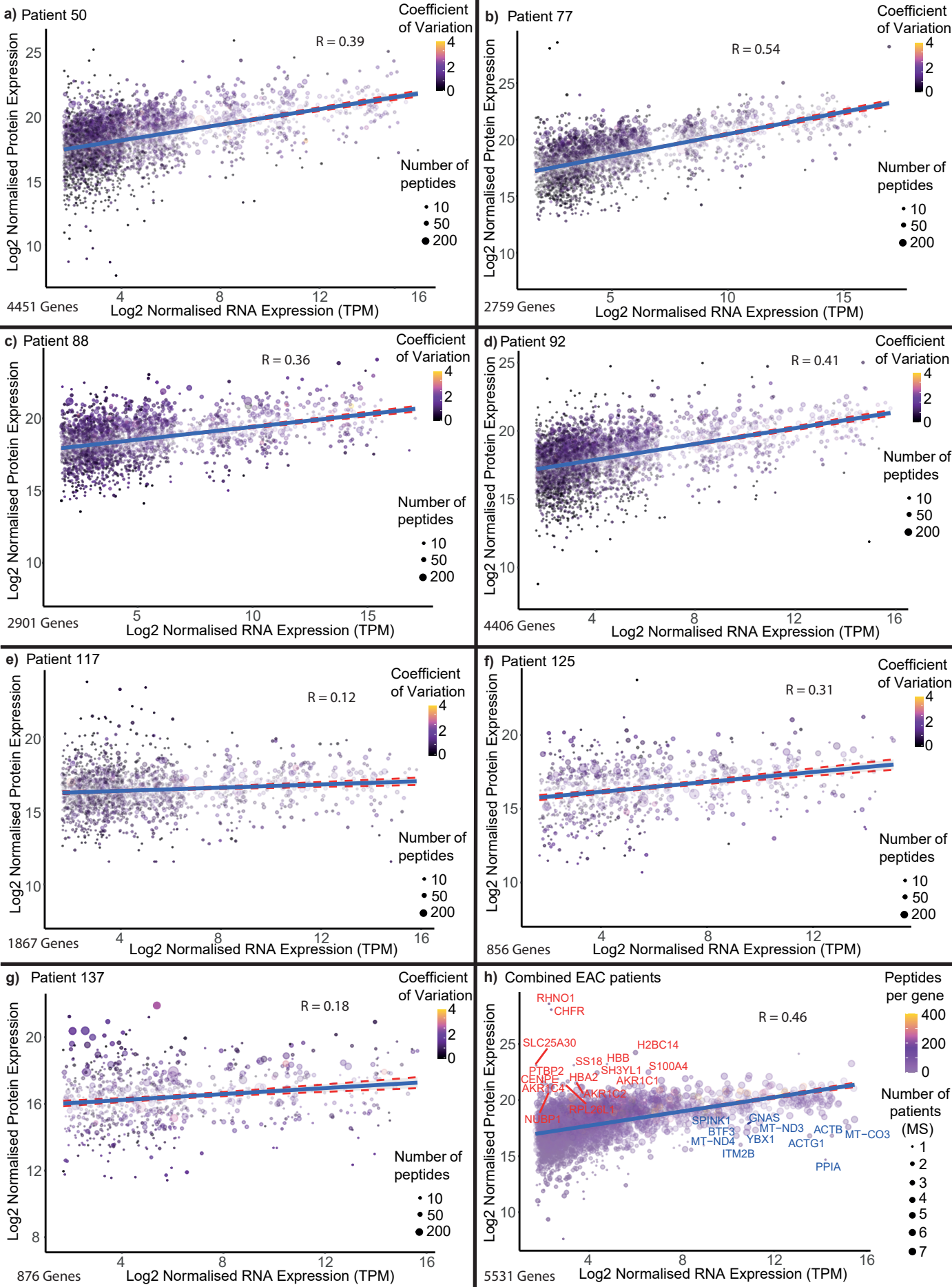

Supplement: Supplemental Figure S6 [file mmc5.pdf]

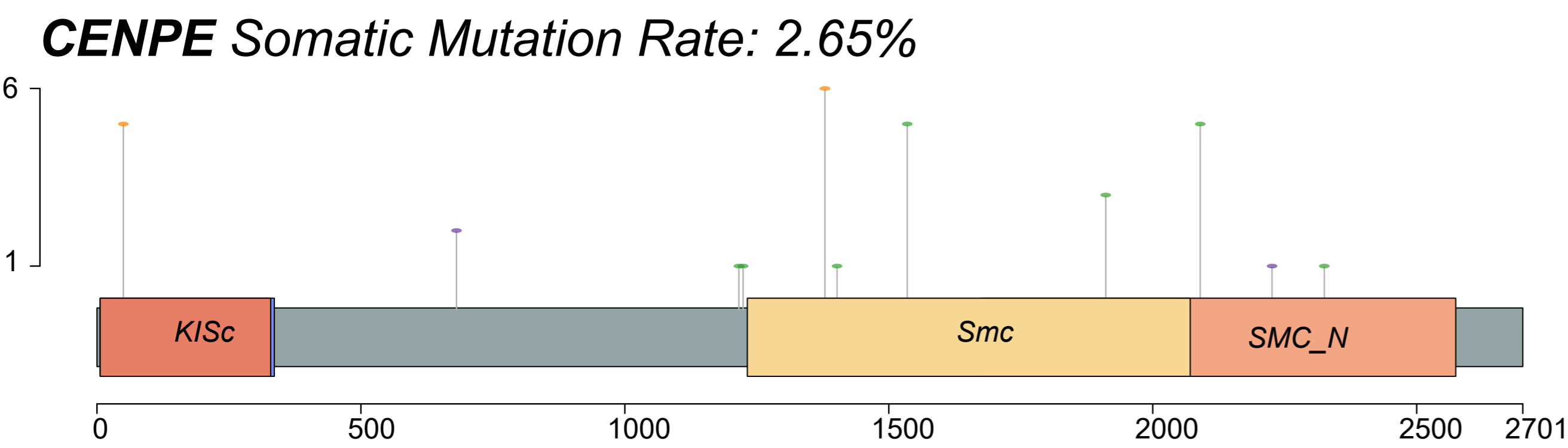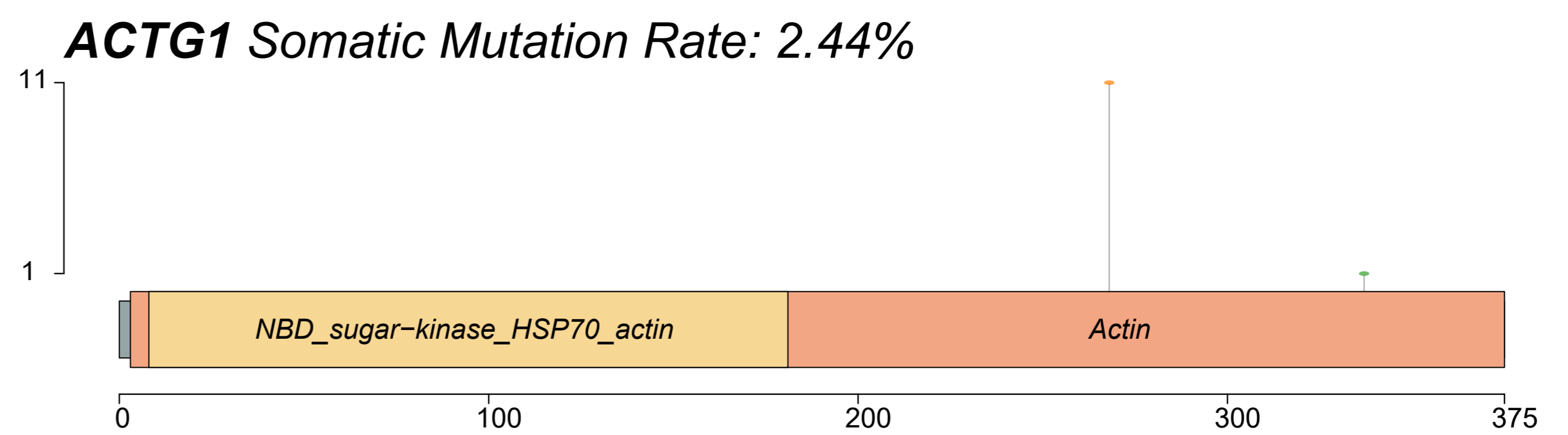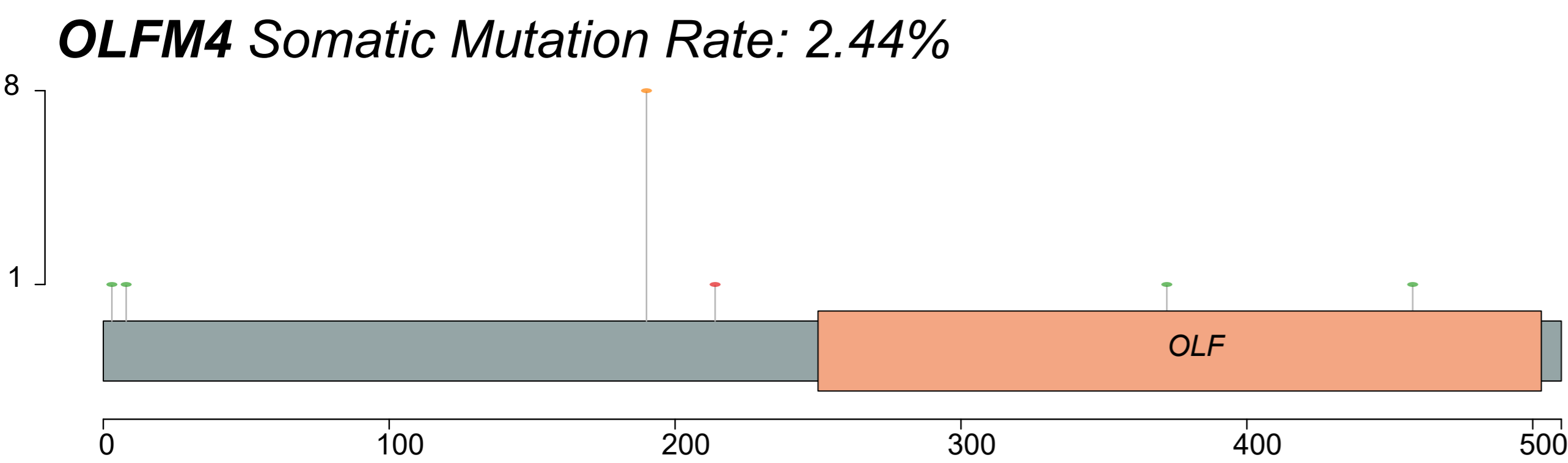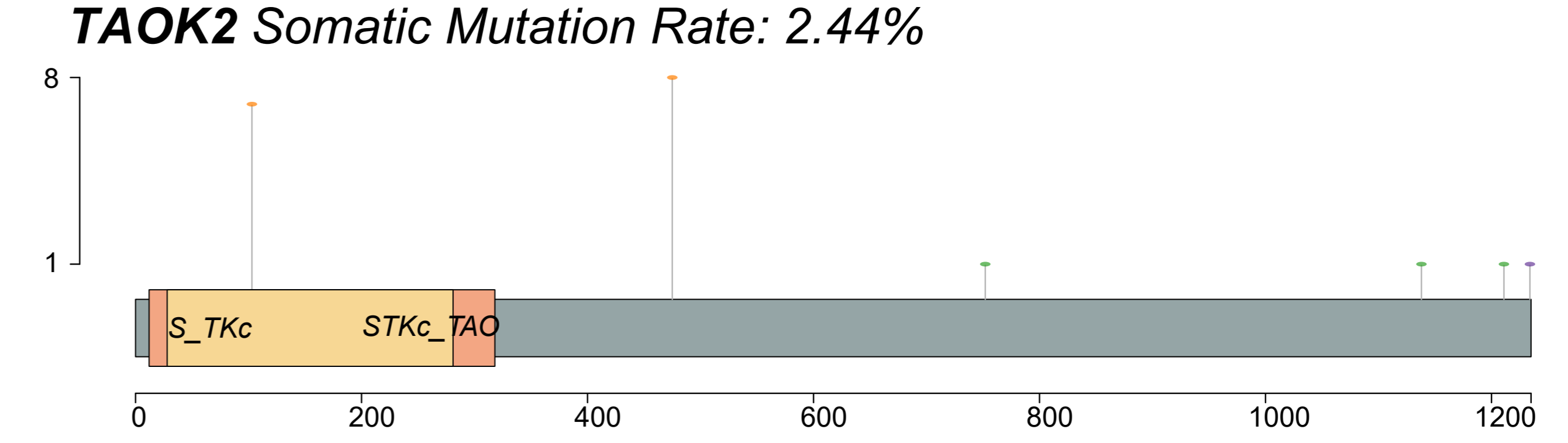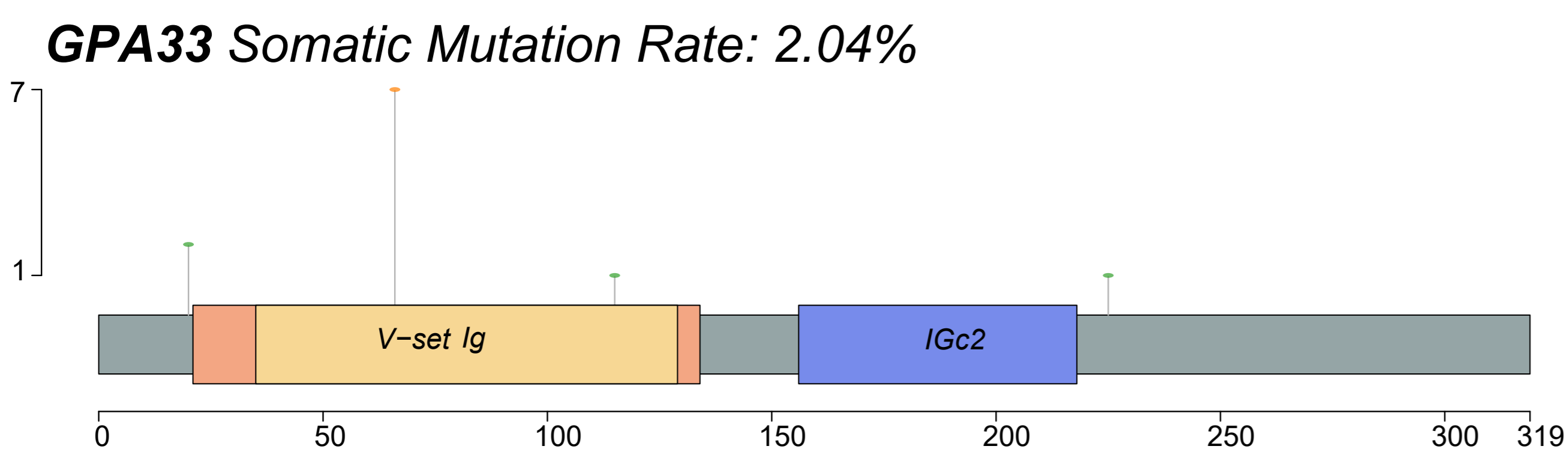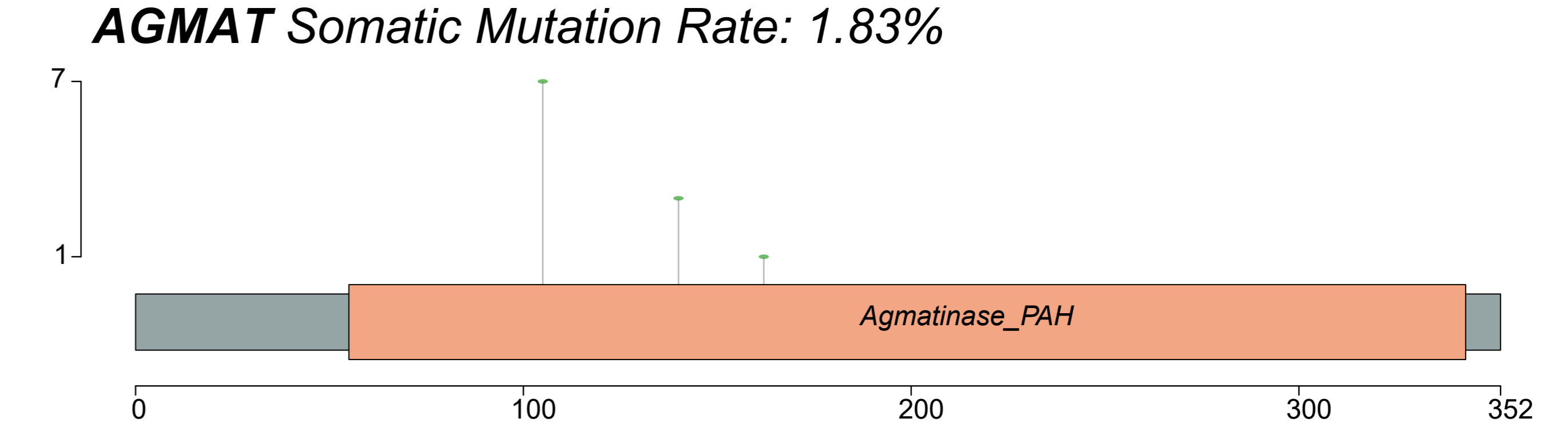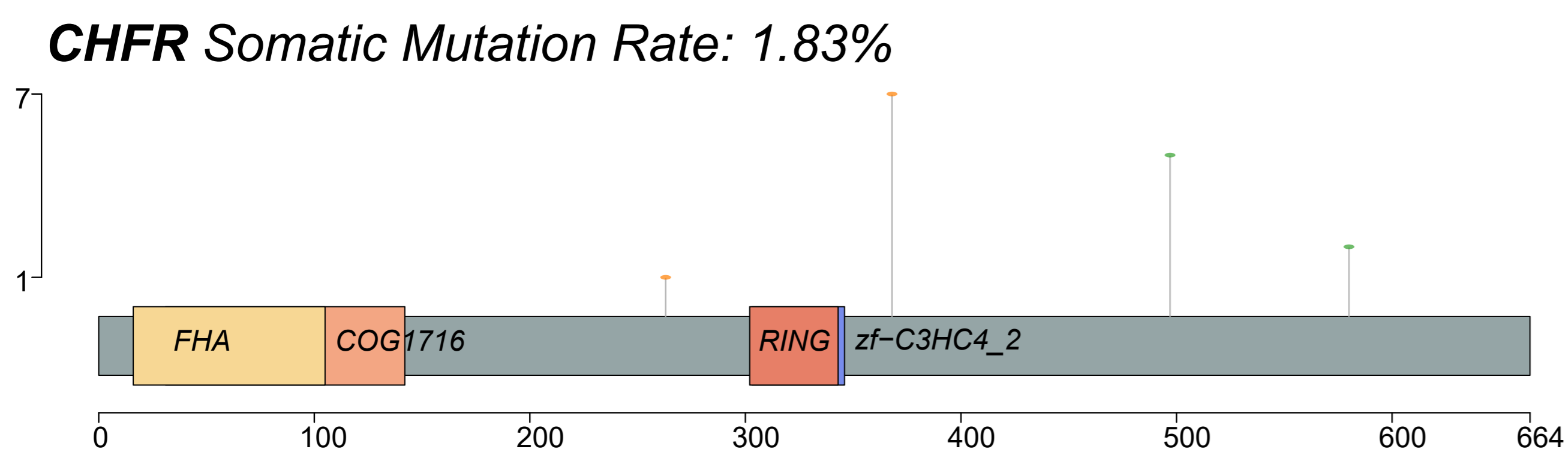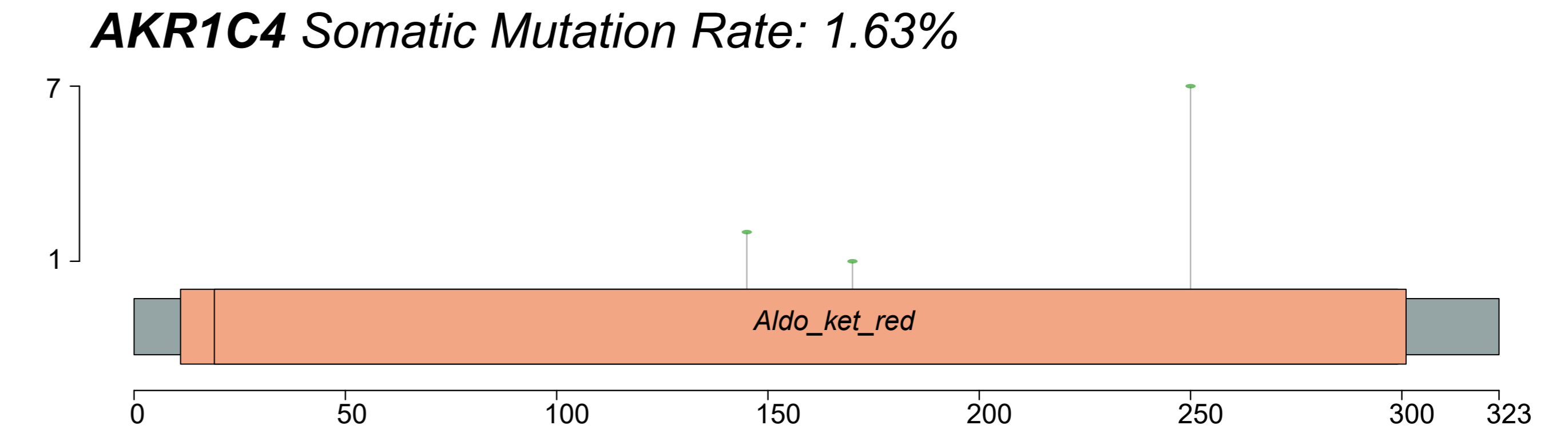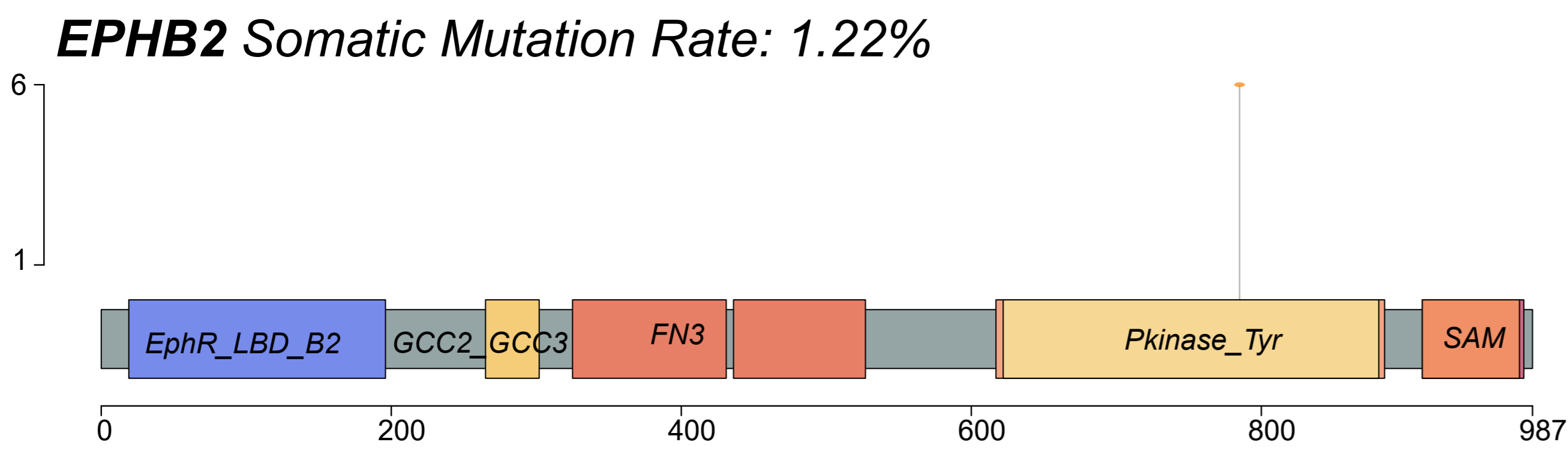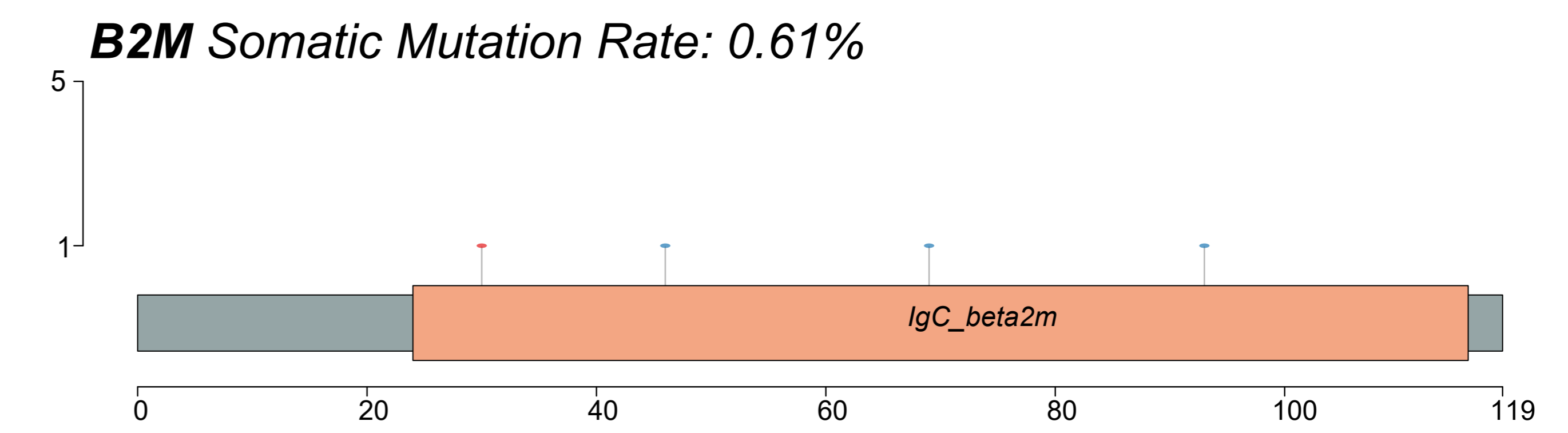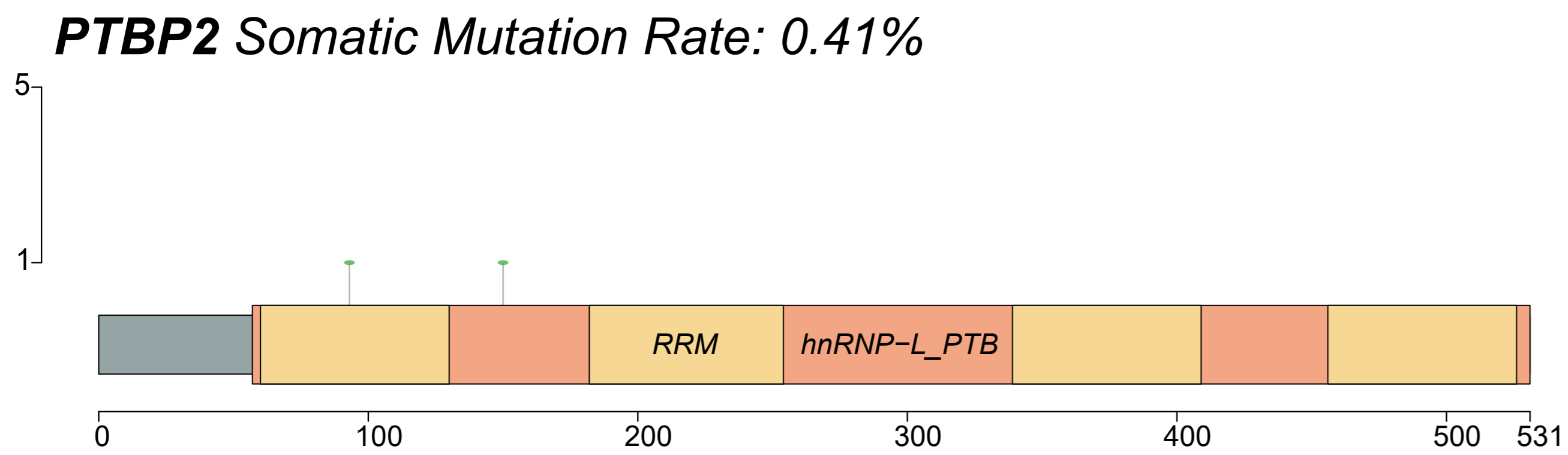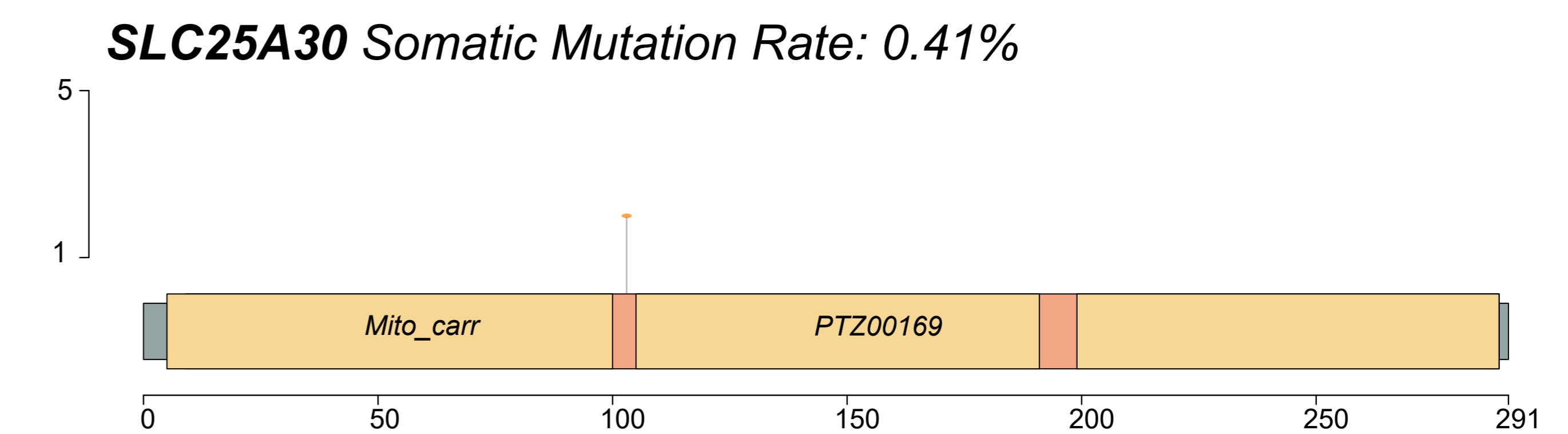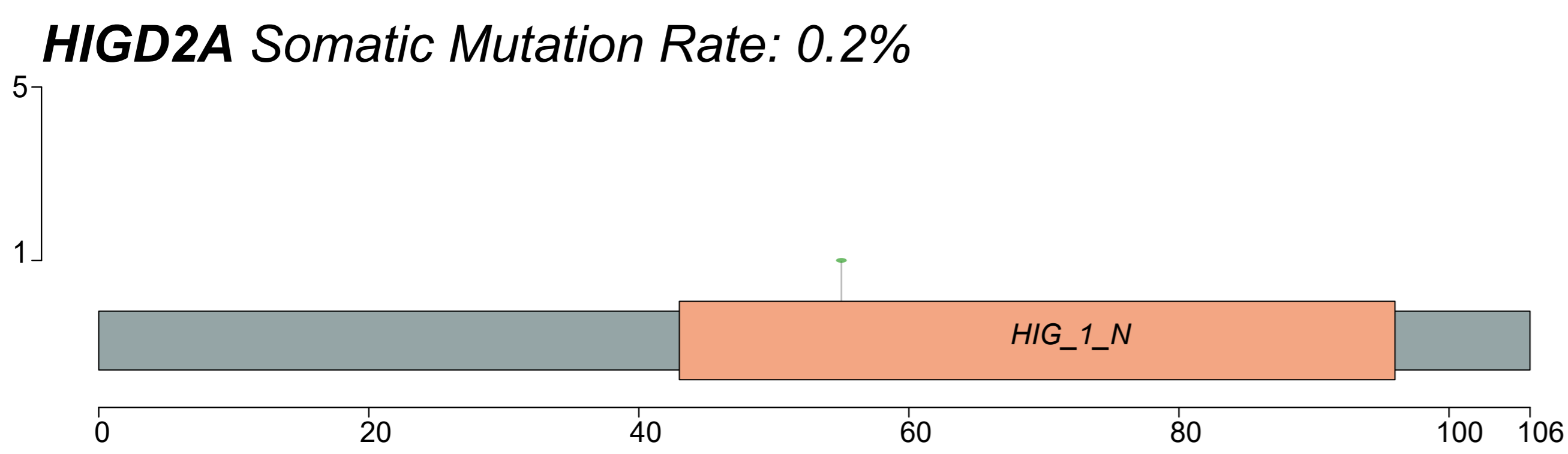

- Splice Site
- Frame Shift Insertion
- Missense Mutation
- Nonsense Mutation
- Frame Shift Deletion

Supplement: Supplemental Figure S7 [file mmc6.pdf]
